# Impact of Hypertrophy‐Oriented Resistance Training on Muscle Mass and Quality of Life in Patients with Unresectable Pancreatic Cancer (HY‐PANC): Study Protocol for a Randomized Controlled Trial

**DOI:** 10.1002/cpz1.70410

**Published:** 2026-07-16

**Authors:** Pablo Braceras Arias, Silvia Burgos Postigo, Lidia B. Alejo

**Affiliations:** ^1^ Department of Sports Sciences, Faculty of Medicine, Health and Sports Research Institute of Hospital 12 de Octubre (imas12) Universidad Europea de Madrid Madrid Spain

**Keywords:** body composition, chemotherapy, lean mass, physical activity, physical function

## Abstract

**Background**: Unresectable pancreatic cancer (PC) is characterized by a high prevalence of sarcopenia and cachexia, which negatively impact survival, treatment toxicity, and quality of life (QoL). Currently, evidence evaluating exercise programs in this vulnerable population is limited.

**Methods**: This prospective, two‐arm randomized controlled trial (RCT) will recruit patients with unresectable PC undergoing chemotherapy. Participants will be randomized (1:1) to an intervention group (hypertrophy‐oriented resistance training twice weekly) or a control group (standard care).

**Discussion**: The primary outcome is the change in total lean body mass (assessed by DXA) at Week 12. Secondary outcomes include maximal strength, peak power, cardiorespiratory capacity, and patient‐reported measures. The HY‐PANC protocol presents a replicable resistance training intervention designed specifically for advanced PC.

**Trial registration**: The protocol has been submitted for review and approval by the Clinical Research Ethics Committee of the University Hospital of Getafe and registered on ClinicalTrials.com (NCT07470268). © 2026 The Author(s). *Current Protocols* published by Wiley Periodicals LLC.

**Abbreviations**
1RM: One Repetition Maximum
PC: Pancreatic Cancer
DXA: Dual‐energy X‐ray Absorptiometry
RCT: Randomized Controlled Trial
ECOG: Eastern Cooperative Oncology Group
EORTC: European Organisation for Research and Treatment of Cancer
HADS: Hospital Anxiety and Depression Scale
HY‐PANC: Hypertrophy‐Oriented Resistance Training in Pancreatic Cancer
BMI: Body Mass Index
ITT: Intention‐to‐Treat
LMM: Linear Mixed Models
QoL: Quality of Life
QLQ: Quality of Life Questionnaire
RPE: Rating of Perceived Exertion
VO2pico: Peak Oxygen Consumption

## INTRODUCTION

### Background and Rationale {6a}

Pancreatic cancer (PC), like all cancers, is a genomic disease caused by abnormalities in the sequence or structure of genomic DNA (Liu et al., 2023). PC remains a highly aggressive neoplasm characterized by a poor prognosis, with a 5‐year survival rate of 13% (Cai et al., 2025, Qiu et al., 2025). Pancreatic ductal adenocarcinoma (PDAC), the most common subtype of PC (de Santiago et al., 2019), is one of the most aggressive and lethal neoplasms (Sarantis et al., 2020), with an extremely unfavorable prognosis (Glapiński et al., 2025, Miller et al., 2016). It is estimated to become the second leading cause of cancer‐related death by 2030 (Sung et al., 2021), and it currently has a 5‐year survival rate close to 13% (Conroy et al., 2018; Kim et al., 2019). Its poor prognosis can be largely explained by late diagnosis and its marked resistance to conventional therapies such as chemotherapy and radiotherapy (Hartwig et al., 2011). Although radical surgery remains the only curative option (Pawlik et al., 2007), up to 80% of patients experience locoregional or distant recurrence after resection, and survival remains low (Jiang et al., 2021; Karunakaran & Barreto, 2021).

In addition to the biological characteristics of the tumor, such as tumor stage or histological grade, physical and nutritional factors are of increasing relevance in determining postoperative prognosis (Lee et al., 2009; Liu et al., 2024). Among them, cachexia (Kern & Norton, 1988) and sarcopenia (Williams et al., 2019) stand out as highly prevalent conditions in PC. Cachexia is a multifactorial syndrome characterized by progressive weight loss, loss of adipose tissue, and muscle mass; it affects around 80% of patients with PDAC (Kordes et al., 2021; Talbert et al., 2019; Tan et al., 2014). Sarcopenia, defined as the loss of muscle mass and function (Ardeljan & Hurezeanu, 2025), is also extremely common throughout the clinical course of PC and shares manifestations with cachexia, such as weakness and systemic inflammation (Evans, 2010), although both nutritional states represent distinct entities (Gupta et al., 2022; Liu et al., 2025).

It has been demonstrated that the presence of cachexia or sarcopenia significantly worsens prognosis (Ashcraft et al., 2019; Cao et al., 2022; Dunne et al., 2019; Larson et al., 2025; Papadopetraki et al., 2023; Yang et al., 2021; Yoh et al., 2018), decreases the response to oncological therapies, increases treatment toxicity, prolongs hospital stays, and deteriorates quality of life (QoL) (Lee et al., 2023; Reed et al., 2019; Spagnolo et al., 2022). Recent studies indicate that both conditions are independent predictors of mortality in multiple cancer types (Owusuaa et al., 2022), especially PC (Rawla et al., 2019), and that preoperative sarcopenia is associated with poorer surgical outcomes in patients undergoing radical resection (Chan & Chok, 2019; di Sebastiano et al., 2013).

As cancer patients have a high risk of functional decline and muscle mass loss (Corrêa & Tabak, 2024; Lavalle et al., 2024), regular aerobic and resistance exercise is recommended to prevent or mitigate these effects (Corrêa et al., 2025; Idorn & Thor Straten, 2017). The scarce evidence suggests that resistance training could potentially be a promising intervention strategy for the prevention and treatment of cancer‐related cachexia and sarcopenia, and consequently an improvement in body composition and physical function in PC patients (Kamel et al., 2020; Lee, 2022; Schmitz et al., 2023).

Overall, understanding the differential impact of sarcopenia and cachexia in unresectable PC patients is essential for optimizing the possible adapted physical interventions (Dalla Via et al., 2021; Shen et al., 2023). Identifying and addressing these conditions through nutritional interventions, specific therapies, and exercise programs could significantly improve clinical outcomes and survival in this highly lethal disease (Dunne et al., 2024).

### Objectives {7}

The main objective of this study is to evaluate the effect on body composition (primarily lean mass) through 12 weeks of hypertrophy training in patients with advanced, non‐operable PC undergoing treatment (chemotherapy and/or immunotherapy). The secondary objectives are as follows:

- To analyze whether 12 weeks of hypertrophy training in patients with advanced, unresectable PC receiving chemotherapy and/or immunotherapy has an impact on cardiorespiratory capacity
- To identify whether 12 weeks of hypertrophy training in patients with advanced, unresectable PC receiving chemotherapy and/or immunotherapy impacts fatigue/asthenia
- To determine whether 12 weeks of hypertrophy training in patients with advanced, unresectable PC receiving chemotherapy and/or immunotherapy has an impact on anxiety and depression

It is hypothesized that the intervention will lead to the maintenance or improvement of muscle mass in patients with advanced unresectable PC, who are undergoing treatment (chemotherapy and/or immunotherapy). Additionally, the same positive impact is expected on cardiorespiratory capacity, QoL, and psychological well‐being aspects such as anxiety and depression.

### Trial Design {8}

The HY‐PANC study protocol features a prospective, two‐arm randomized controlled trial (RCT) design. The intervention will be carried out at the facilities of Universidad Europea de Madrid in Villaviciosa de Odón, and the participants will be recruited from Hospital Universitario Puerta de Hierro (HUPR). Prior to enrollment, all eligible participants will receive a detailed participant information sheet (PIS) and will be required to sign the informed consent form (ICF) in accordance with ethical and regulatory standards.

## METHODS: PARTICIPANTS, INTERVENTIONS, AND OUTCOMES

### Study Setting {9}

Demographics and clinical characteristics will be collected at one public hospital in Madrid, Spain. HUPR will collect information regarding birth date, sex, participant code, height, and weight. Additionally, detailed information regarding the chemotherapy regimen will be collected, including treatment type (e.g., FOLFIRINOX or gemcitabine‐based regimens), treatment line, and cycle number at study entry.

### Eligibility Criteria {10}

Patients will be eligible if they meet the following inclusion criteria: a diagnosis of locally advanced or metastatic unresectable PC, undergoing chemotherapy or immunotherapy, age ≥ 18 years, and ECOG score ≤ 2. The exclusion criteria will be any relative or absolute contraindication that prevents the performance of training or physical tests, diagnosis of another type of primary cancer, or the inability to understand Spanish for proper comprehension of the informed consent and questionnaires.

### Who will take Informed Consent? {26a}

All eligible participants will receive a PIS and must sign the ICF prior to enrollment.

### Additional Consent Provisions for Collection and Use of Participant Data and Biological Specimens {26b}

#### Images/video

There is a specific additional consent provision for recording images or videos of participants during physical tests or training sessions for scientific and educational purposes.

#### Biological specimens

The protocol does not mention the collection of biological specimens (e.g., blood or tissue) requiring additional consent; it focuses on physical and body composition measurements.

## INTERVENTIONS

### Explanation for the Choice of Comparators {6b}

The control group will receive standard oncological care without physical activity restrictions to compare the efficacy of the added intervention.

### Intervention Description {11a}

Participants in the intervention group will receive their standard oncological treatment and, in addition, will complete a 12‐week hypertrophy training program. Before these 12 weeks, they will undergo a 2‐week familiarization period with the program's exercises. The structure of the intervention is as follows:

Twice per week, in 80‐minute sessions, participants will perform a light‐to‐moderate‐intensity aerobic warm‐up with joint mobility exercises, followed by a main block of strength exercises focused on hypertrophy, targeting the major muscle groups. During the 2‐week familiarization period, a high number of repetitions and low‐intensity loads will be used (2 sets of 10–15 repetitions per exercise). Once the intervention begins, both volume and intensity will increase to 2 or 3 sets and from 10–15 to 5–6 repetitions. The selected loading scheme (5–6 repetitions) was chosen to balance mechanical tension with tolerability and safety in a clinically vulnerable population.

Intensity will be monitored using the Rate of Perceived Exertion (RPE) scale, with the goal of consistently exceeding moderate intensity in each exercise performed. Progression will be adapted individually for each participant. All sessions will be supervised by professionals in physical activity and sport sciences. Sessions will take place in group format at the facilities of the Universidad Europea de Madrid in Villaviciosa de Odón. Table 1 provides a detailed description of the intervention components. Table 2 presents the participant timeline.

**Table 1:** Description of the Supervised Exercise Program

Duration of exercise program	2 weeks of familiarization (4 sessions) + 12 weeks of intervention (24 sessions)
Duration of exercise sessions	80 min
Exercise frequency	2 weekly sessions
Rest period between exercise sessions	2 days
Warm‐up	Light‐to‐moderate‐intensity aerobic exercise (stationary bike) and joint mobility work of the shoulder, hip, knee, ankle, and shoulder and pelvic girdle joints
Descriptions of the resistance exercises	Push, pull, knee predominant, hip predominant, and core
Number of repetitions/ intensity (RPE)	10–15/RPE 5 (the first 4 familiarization sessions), 5–6/RPE 7 (in the 24 intervention sessions)
Number of sets per session	2 series (the first 4 familiarization sessions), 3 series (in the 24 intervention sessions)
Rest period between sets	60 s

**Table 2:** Participant Timeline

HY‐PANC STUDY TIMELINE					
	**Enrollment**	**Allocation**	**Post‐allocation (intervention)**	**Close‐out**	
**Time point**	**Week ‐2**	**Week 0**	**Weeks 1–2** *(Familiarization)*	**Weeks 3–14** *(Training)*	**Week 14** *(Post‐test)*
**Enrollment**:					
Eligibility screen	**X**				
Informed consent	**X**				
Medical history and ECOG	**X**				
Allocation		**X**			
Sarcopenia/cachexia assessment		**X**			**X**
Chemotherapy regimen and treatment data	**X**	**X**	**X**	**X**	**X**
**Interventions**:					
**Intervention group** *(hypertrophy resistance training)*			**X**	**X**	
**Control group** *(standard oncological care)*			**X**	**X**	
**Assessments**:					
**Sociodemographic data**	**X**				
**Anthropometry** (height, weight, and BMI)		**X**			**X**
**Body composition** (DXA ‐ lean mass)		**X**			**X**
**Maximal strength** (1RM estimation)		**X**			**X**
**Cardiorespiratory fitness** (VO2peak)		**X**			**X**
**Muscle power** (6‐second test)		**X**			**X**
**Quality of life** (EORTC QLQ‐C30/PAN26)		**X**			**X**
**Anxiety and depression** (HADS)		**X**			**X**
**Fatigue** (MFI/visual scale) *		**X**			**X**
**Adverse events/adherence**			**X**	**X**	

Participants in the control group will receive standard oncological treatment. No restrictions on physical activity will be imposed. Furthermore, they will be offered the opportunity to follow the exercise program used in the intervention group if the expected benefits proposed in the study hypothesis are confirmed.

### Criteria for Discontinuing or Modifying Allocated Interventions {11b}

Discontinuation criteria: acute symptoms (dizziness, chest pain), excessive effort (RPE > 9), oncological contraindications (fever > 38°C, uncontrolled pain), or clinical deterioration (ECOG > 2)

### Strategies to Improve Adherence to Interventions {11c}

Sessions are supervised by professionals. Intention‐to‐treat (ITT) analysis includes everyone regardless of adherence, but attendance will be recorded.

### Relevant Concomitant Care Permitted or Prohibited During the Trial {11d}

Patients continue with their standard oncological treatment (chemotherapy/immunotherapy). No physical activity restrictions are imposed on the control group.

### Provisions for Post‐Trial Care {30}

The control group will be offered the opportunity to follow the exercise program if the benefits are confirmed.

### Outcomes {12}

Primary outcome: Change in total lean body mass (assessed by DXA) at week 12

Secondary outcomes: Maximal strength (1RM estimation), peak oxygen consumption (VO2peak), lower limb peak power, QoL (EORTC, QLQ‐C30, and QLQ‐PAN26), anxiety, and depression (HADS)

#### Exploratory outcomes: Assessment of sarcopenia and cachexia

Sarcopenia will be defined according to the revised European Working Group on Sarcopenia in Older People criteria (EWGSOP2), incorporating measures of muscle mass (DXA‐derived lean mass) and muscle strength (1RM estimation).

Cancer cachexia will be defined according to the international consensus by Fearon et al., based on weight loss (>5% over the previous 6 months), BMI, and/or reduced muscle mass.

Baseline prevalence and changes over the 12‐week intervention period will be analyzed descriptively.

#### Chemotherapy‐related outcomes

The following variables will be collected to assess treatment tolerability:

‐ Relative Dose Intensity (RDI)
‐ Dose reductions (%)
‐ Treatment delays
‐ Treatment completion rates
‐ Chemotherapy‐related adverse events (graded according to CTCAE v5.0)

These outcomes will be analyzed as exploratory endpoints.

### Participant Timeline {13}

#### Sample size {14}

The sample size was calculated based on the primary outcome: change in total lean body mass assessed by DXA.

Based on previous studies in oncology populations undergoing resistance training, we assumed a mean decline of 1.0 kg in the control group and a mean increase of 0.5 kg in the intervention group, resulting in an expected between‐group difference of 1.5 kg. This difference was considered clinically meaningful (minimal clinically important difference, MCID).

A standard deviation (SD) of 1.5 kg was assumed based on prior literature in comparable populations.

The calculation was performed using G*Power software (version 3.1.9.7) for an F‐test (ANOVA: repeated measures, within‐between interaction), with the following parameters:

‐ Significance level (α): 0.05 (two‐tailed)
‐ Statistical power (1‐β): 0.80
‐ Number of groups: 2
‐ Number of measurements: 2 (baseline and post‐intervention)
‐ Correlation between repeated measures: 0.5

The analysis indicated that a minimum total sample size of 34 participants (17 per group) is required. To account for an expected attrition rate of 20%–25%, a total of 44 participants (22 per group) will be recruited.

#### Recruitment {15}

Participants will be recruited at the University Hospital Puerta de Hierro.

### Assignment of Interventions: Allocation

#### Sequence generation {16a}

A 1:1 block randomization will be generated using specialized software (sealed envelope).

#### Concealment mechanism {16b}

A centralized password‐protected web‐based system will be used (sealed envelope). Allocation is concealed from recruitment researchers until eligibility is confirmed and baseline data are uploaded.

#### Implementation {16c}

The software assigns participants automatically. The physical assessment researcher remains blinded to the assignment. Notifications are sent directly to the exercise supervision team.

### Assignment of Interventions: Blinding

#### Who will be blinded {17a}

This is a single‐blind study (assessor blinded). Patients and trainers cannot be blinded. Outcome assessors (DXA technicians, 1RM analysts, and statisticians) will be blinded.

### Procedure for Unblinding if Needed {17b}

The group code will only be revealed after the main statistical analysis is finalized.

### Data Collection and Management

#### Plans for assessment and collection of outcomes {18a}

*Physical*: 1RM test (force‐velocity methodology), incremental cycle ergometer test (VO2peak), and 6‐second test (power)

*Body*: Weight, height, and body composition via DXA

*Questionnaires*: EORTC QLQ‐C30, QLQ‐PAN26, and HADS

### Plans to Promote Participant Retention and Complete Follow‐up {18b}

- Intention‐to‐Treat (ITT) analysis
- Exhaustive recording of reasons for dropout

### Data Management {19}

- Pseudonymization with identification codes (e.g., HYPANC‐001)
- Separate and protected master list
- Electronic data on encrypted institutional drives
- Physical records under lock and key
- Storage for 5 years

### Confidentiality {27}

- Compliance with GDPR and LOPD‐GDD
- Use of pseudonymized codes to ensure anonymity

### Plans for Collection, Laboratory Evaluation, and Storage of Biological Specimens for Genetic or Molecular Analysis in this Trial/Future Use {33}

No specific biological specimens other than non‐invasive body measurements are mentioned in this protocol.

### Statistical Methods

#### Statistical methods for primary and secondary outcomes {20a}

All statistical analyses will be performed using SPSS Statistics software (version 28.0 or later; IBM Corp.). The significance level will be set at p < 0.05 for all tests. The normality of the data distribution will be assessed using the Shapiro–Wilk test and visual inspection of histograms. Continuous variables with a normal distribution will be presented as mean ± SD. Non‐normally distributed variables will be reported as median and interquartile range (IQR). Categorical variables (e.g., gender, tumor stage, and ECOG) will be expressed as frequencies (n) and percentages (%).

#### Main analysis (primary and secondary outcomes)

To determine the efficacy of the intervention, an ITT analysis will be conducted, including all randomized participants regardless of adherence.

#### Linear mixed models (LMMs)

The effects of the training program on body composition (muscle mass), physical function (1RM, VO_2_peak), and QoL will be analyzed using LMMs.

*Fixed effects*: Group (Intervention vs. Control), Time (Baseline vs. 12 weeks), Group × Time interaction, and chemotherapy regimen as a covariate.

*Random effects*: Participants will be included as a random effect to account for individual variability and baseline differences.

This method is preferred over ANOVA as it handles missing data (common in oncology trials) more robustly without the need for imputation.

#### Secondary analyses

*Per‐protocol analysis*: An additional sensitivity analysis will be performed, including only those participants who completed >75% of the supervised sessions.

*Baseline comparisons*: Potential baseline differences between groups will be assessed using independent t‐tests (for continuous normal data) or Mann–Whitney U tests (for non‐normal data). Chi‐square tests (χ²) will be used for categorical variables.

#### Interim analyses {21b}

No interim analyses are explicitly planned. The group assignment code will only be revealed after the primary statistical analysis is finalized.

### Methods for Additional Analyses (e.g., Subgroup Analyses) {20b}

#### Per‐protocol analysis

A sensitivity analysis will be performed, including only those participants who completed >75% of the supervised sessions, to assess the efficacy of the intervention in adherent patients.

#### Baseline comparisons

Potential baseline differences between groups will be assessed using independent t‐tests (for continuous normal data), Mann‐Whitney U tests (for non‐normal data), and chi‐square tests (χ²) for categorical variables.

### Methods in Analysis to Handle Protocol Non‐adherence and any Statistical Methods to Handle Missing Data {20c}

#### Protocol non‐adherence

The primary analysis will be ITT, including all randomized participants regardless of adherence. Additionally, a per‐protocol analysis will be conducted for participants with >75% adherence.

#### Missing data

LMMs will be used for the analysis, as this method handles missing data—common in oncology trials—more robustly without the need for imputation.

### Plans to Give Access to the Full Protocol, Participant‐Level Data, and Statistical Code {31c}

Access to participant‐level data is restricted to the research team and authorized personnel to guarantee confidentiality. Data will be stored on encrypted institutional drives, and physical records will be kept under lock and key for a minimum of 5 years, after which they will be securely destroyed. No specific plans for public access to the full protocol or statistical code are mentioned in the protocol.

### Oversight and Monitoring

#### Composition of the coordinating center and trial steering committee {5d}

Sessions will be supervised by professionals in sports and physical activity sciences.

#### Composition of the data monitoring committee, its role, and reporting structure {21a}

A formal external Data Monitoring Committee (DMC) is not detailed, but strict safety supervision is in place.

#### Adverse event reporting and harms {22}

- Strict safety and termination criteria
- Civil liability insurance contracted
- Recording of dropouts due to toxicity or disease progression

#### Frequency and plans for auditing trial conduct {23}

Not specified

### Plans for Communicating Important Protocol Amendments to Relevant Parties (e.g., Trial Participants and Ethical Committees) {25}

The protocol does not explicitly detail a formal procedure for communicating protocol amendments to investigators, the REC, or registries. However, it states that the study observes strict adherence to ethical standards and regulatory compliance, implying amendments would likely follow standard regulatory channels (e.g., ethics committee approval), though this is not written in the protocol.

### Dissemination Plans {31a}

The study plans for scientific dissemination through congresses, academic papers, and teaching activities. The results will be published in reports or presentations, ensuring that participants' names never appear. The protocol aims to provide Level I evidence to support integrating this training into standard oncological care.

## DISCUSSION

The HY‐PANC protocol presents details of a prospective RCT designed to investigate the effects of a 12‐week, hypertrophy‐oriented strength training program on patients with unresectable advanced PC undergoing treatment. This study is critically important given the extremely aggressive nature and poor prognosis of PC, compounded by the high prevalence of cancer‐related cachexia and sarcopenia. The presence of these conditions significantly worsens prognosis, reduces therapeutic response, and compromises the QoL. The core rationale of this work is to address the recognized need for interventions, such as resistance exercise, to prevent or mitigate functional decline and muscle loss in this population. The elements of exercise in the intervention are designed in accordance with international recommendations for exercise training in healthy individuals and in patients with cancer (American College of Sports Medicine, 2009; Schmitz et al., 2010). Additionally, this study incorporates exploratory analyses of sarcopenia and cachexia, as well as chemotherapy‐related outcomes, to better understand the clinical and translational relevance of resistance training in this population.

Although contemporary guidelines advocate for the inclusion of resistance training in oncology care (Fairman, 2024), the evidence is notably scarce, particularly for patients with advanced PC (Rosebrock et al., 2023). The current protocol is distinguished by several novel elements that address gaps in the existing literature: the use of gold standard and validated methodologies (DXA, VO_2_ peak via incremental cycle ergometry, EORTC QLQ‐C30, and the PC‐specific QLQ‐PAN26). The intervention employs a structured, supervised, and progressive training model specifically targeting muscle hypertrophy, with a specific focus on patients with non‐operable PC.

Despite the rigorous design, this trial is subject to several anticipated limitations inherent to oncology research: The aggressive nature of advanced PC may pose challenges to patient recruitment and retention over the 12‐week intervention period.

If the hypothesis that hypertrophy training leads to the maintenance or improvement of muscle mass, function, and QoL in this high‐risk PC cohort is confirmed, this RCT has the potential to provide Level I evidence (Burns et al., 2011) supporting the integration of supervised, high‐intensity resistance training into standard oncological care for advanced PC. Such a finding would not only validate exercise as a potent supportive care strategy against cachexia/sarcopenia but could fundamentally shift the paradigm toward prescribing targeted exercise as an “onco‐therapeutic” intervention. Future research should focus on optimizing exercise dosing (frequency, intensity, and time) and exploring the synergistic effects of combining this training protocol with advanced nutritional interventions.

The HY‐PANC study protocol represents a replicable and safe strength‐oriented intervention designed specifically for the clinical challenges of advanced PC. By addressing the critical prevalence of sarcopenia and cachexia, this study has the potential to provide Level I evidence supporting the integration of supervised resistance training into standard oncological care.

Unlike general physical activity advice, this protocol provides a specific dosage and methodology that could fundamentally improve the prognosis, therapeutic response, and QoL for patients facing this aggressive disease. If effective, HY‐PANC will serve as a pioneering model for high‐intensity supportive care in advanced gastrointestinal oncology.

## Trial status

Recruiting

### Author Contributions {31b}

**Pablo Braceras Arias**: Investigation; conceptualization; methodology. **Silvia Burgos Postigo**: Conceptualization. **Lidia B. Alejo**: Conceptualization.

### Conflict of Interest {28}

The authors declare no conflict of interest.

## Ethics Approval and Consent to Participate {24}

The protocol has been submitted for review and approval by the Clinical Research Ethics Committee of the Getafe University Hospital. Participation is voluntary, and all eligible participants must sign a written informed consent form prior to enrollment after receiving the Participant Information Sheet.

## Consent for Publication {32}

Specific consent is sought for the use of images or videos for scientific dissemination (e.g., in congresses and papers). The form specifies that faces will be blurred or otherwise obscured to protect identity whenever possible, and that names will not be associated with the images. Participants can choose to authorize or not authorize this use without affecting their participation in the main study.

## Informed consent for participation

Project Title: HY‐PANC Study Principal Investigator: Pablo Braceras Arias

## Statement of the Participant

1. I have read the Participant Information Sheet regarding the study “Impact of Hypertrophy‐Oriented Strength Training in patients with unresectable PC.”

2. I have had the opportunity to ask questions about the study, and these have been answered to my satisfaction.

3. I understand that my participation is voluntary and that I am free to withdraw at any time, without giving any reason, and without my medical care or legal rights being affected.

4. I understand that my medical notes and data collected during the study may be looked at by responsible individuals from the research team or regulatory authorities (where relevant to my participation in this research). I give permission for these individuals to have access to my records.

5. I consent to the processing of my personal data for the purposes of this research, in accordance with the GDPR and LOPD‐GDD.

## I agree to take part in the above study

**Participant**: Name:

________________________________________________ Signature:

_____________________________________________ Date: **/**/202_

**Researcher**: Name:

________________________________________________ Signature:

_____________________________________________ Date: **/**/202_

## Informed consent for image publication

Scientific dissemination (e.g., congresses, academic papers, and teaching) may require the use of images or videos to illustrate the exercise technique or physical progress.

I, Mr./Ms. _________________________________________, hereby authorize the HY‐PANC research team to:

1. Record images or videos of me during the physical tests or training sessions.

2. Use these images for strictly scientific and educational purposes.

I understand that:

• My face will be blurred or obscured whenever possible to protect my identity.

• My name will never be associated with the images; a code will be used.

• I can revoke this authorization at any time without affecting my participation in the main study.

Please check one box: [ ] **I AUTHORIZE** the recording and use of my images for scientific purposes. [ ] **I DO NOT AUTHORIZE** (I can still participate in the study without being recorded).

**Participant's Signature**: ______________________ **Date**: //202_

## Data Availability

Access to data is restricted to the research team and authorized personnel to guarantee confidentiality. Data will be stored for a minimum of 5 years and then securely destroyed. There is no statement regarding the public availability of the dataset (e.g., “upon reasonable request”).

## References

[cpz170410-bib-0002] American College of Sports Medicine . (2009). American College of Sports Medicine position stand. Progression models in resistance training for healthy adults. Medicine & Science in Sports & Exercise, 41(3), 687–708. https://10.1249/MSS.0b013e3181915670 19204579 10.1249/MSS.0b013e3181915670

[cpz170410-bib-0003] Ardeljan, A. D. , & Hurezeanu, R. (2025). Sarcopenia. In: StatPearls [Internet]. Treasure Island, FL: StatPearls Publishing. http://www.ncbi.nlm.nih.gov/books/NBK560813/

[cpz170410-bib-0004] Ashcraft, K. A. , Warner, A. B. , Jones, L. W. , & Dewhirst, M. W. (2019). Exercise as adjunct therapy in cancer. Seminars in Radiation Oncology, 29(1), 16–24. 10.1016/j.semradonc.2018.10.001 30573180 PMC6656408

[cpz170410-bib-0006] Burns, P. B. , Rohrich, R. J. , & Chung, K. C. (2011). The levels of evidence and their role in evidence‐based medicine. Plastic and Reconstructive Surgery, 128(1), 305–310. https://10.1097/PRS.0b013e318219c171 21701348 10.1097/PRS.0b013e318219c171 PMC3124652

[cpz170410-bib-0007] Cai, F. , Gu, Y. , Ling, Y. , Yi, G. , Zang, S. , Su, T. , Liu, Y. , Li, A. , Wang, D. , Zhao, W. , Xie, X. , Li, G. , Dai, L. , Gong, M. , Yang, H. , Zhao, Y. , & Zhang, Y. (2025). Proteomics in pancreatic cancer. Biomarker Research, 13(1), 93. 10.1186/s40364-025-00805-y 40619414 PMC12232871

[cpz170410-bib-0009] Cao, A. , Ferrucci, L. M. , Caan, B. J. , & Irwin, M. L. (2022). Effect of exercise on sarcopenia among cancer survivors: A systematic review. Cancers, 14(3), 786. 10.3390/cancers14030786 35159052 PMC8833677

[cpz170410-bib-0010] Chan, M. Y. , & Chok, K. S. H. (2019). Sarcopenia in pancreatic cancer — effects on surgical outcomes and chemotherapy. World Journal of Gastrointestinal Oncology, 11(7), 527–537. http://10.4251/wjgo.v11.i7.527 31367272 10.4251/wjgo.v11.i7.527 PMC6657219

[cpz170410-bib-0012] Conroy, T. , Hammel, P. , Hebbar, M. , Ben Abdelghani, M. , Wei, A. C. , Raoul, J.‐L. , Choné, L. , Francois, E. , Artru, P. , Biagi, J. J. , Lecomte, T. , Assenat, E. , Faroux, R. , Ychou, M. , Volet, J. , Sauvanet, A. , Breysacher, G. , di Fiore, F. , Cripps, C. , … Bachet, J.‐B. (2018). FOLFIRINOX or gemcitabine as adjuvant therapy for pancreatic cancer. New England Journal of Medicine, 379(25), 2395–2406. 10.1016/j.acthis.2026.152349 30575490

[cpz170410-bib-0013] Corrêa, R. , de Almeida, C. V. , da Fonseca, V. , & Tabak, B. M. (2025). Influence of physical exercise on health improvement in older adult cancer patients: A systematic review. Frontiers in Public Health, 13, 1525021. 10.3389/fpubh.2025.1525021 40860535 PMC12375496

[cpz170410-bib-0014] Corrêa, R. , & Tabak, B. M. (2024). The influence of behavioral sciences on adherence to physical activity and weight loss in overweight and obese patients: A systematic review of randomized controlled trials. International Journal of Environmental Research and Public Health, 21(5), 630. 10.3390/ijerph21050630 38791844 PMC11121225

[cpz170410-bib-0015] Dalla Via, J. , Owen, P. J. , Daly, R. M. , Mundell, N. L. , Livingston, P. M. , Rantalainen, T. , Foulkes, S. J. , Millar, J. L. , Murphy, D. G. , & Fraser, S. F. (2021). Musculoskeletal responses to exercise plus nutrition in men with prostate cancer on androgen deprivation: A 12‐month RCT. Medicine & Science in Sports & Exercise, 53(10), 2054–2065. 10.1249/mss.0000000000002682 33867499

[cpz170410-bib-0016] de Santiago, I. , Yau, C. , Heij, L. , Middleton, M. R. , Markowetz, F. , Grabsch, H. I. , Dustin, M. L. , & Sivakumar, S. (2019). Immunophenotypes of pancreatic ductal adenocarcinoma: Meta‐analysis of transcriptional subtypes. International Journal of Cancer, 145(4), 1125–1137. 10.1002/ijc.32186 30720864 PMC6767191

[cpz170410-bib-0017] di Sebastiano, K. M. , Yang, L. , Zbuk, K. , Wong, R. K. , Chow, T. , Koff, D. , Moran, G. R. , & Mourtzakis, M. (2013). Accelerated muscle and adipose tissue loss may predict survival in pancreatic cancer patients: The relationship with diabetes and anaemia. British Journal of Nutrition, 109(2), 302–312. 10.1017/S0007114512001067 23021109

[cpz170410-bib-0018] Dunne, R. F. , Crawford, J. , Smoyer, K. E. , McRae, T. D. , Rossulek, M. I. , Revkin, J. H. , Tarasenko, L. C. , & Bonomi, P. D. (2024). The mortality burden of cachexia or weight loss in patients with colorectal or pancreatic cancer: A systematic literature review. Journal of Cachexia, Sarcopenia and Muscle, 15(5), 1628–1640. 10.1002/jcsm.13510 39095951 PMC11446707

[cpz170410-bib-0019] Dunne, R. F. , Loh, K. P. , Williams, G. R. , Jatoi, A. , Mustian, K. M. , & Mohile, S. G. (2019). Cachexia and sarcopenia in older adults with cancer: A comprehensive review. Cancers, 11(12), 1861. 10.3390/cancers11121861 31769421 PMC6966439

[cpz170410-bib-0020] Evans, W. J. (2010). Skeletal muscle loss: Cachexia, sarcopenia, and inactivity123. The American Journal of Clinical Nutrition, 91(4), 1123S–1127S. 10.3945/ajcn.2010.28608A 20164314

[cpz170410-bib-0021] Fairman, C. M. (2024). A practical framework for the design of resistance exercise interventions in oncology research settings—a narrative review. Frontiers in Sports and Active Living, 6, 1418640. 10.3389/fspor.2024.1418640 39703544 PMC11655215

[cpz170410-bib-0023] Glapiński, F. , Zając, W. , Fudalej, M. , Deptała, A. , Czerw, A. , Sygit, K. , Kozłowski, R. , & Badowska‐Kozakiewicz, A. (2025). The role of the tumor microenvironment in pancreatic ductal adenocarcinoma: Recent advancements and emerging therapeutic strategies. Cancers, 17(10), 1599. 10.3390/cancers17101599 40427098 PMC12110676

[cpz170410-bib-0024] Gupta, P. , Hodgman, C. F. , Schadler, K. L. , & LaVoy, E. C. (2022). Effect of exercise on pancreatic cancer patients during treatment: A scoping review of the literature. Supportive Care in Cancer, 30(7), 5669–5690. 10.1007/s00520-022-06925-7 35190894

[cpz170410-bib-0025] Hartwig, W. , Hackert, T. , Hinz, U. , Gluth, A. , Bergmann, F. , Strobel, O. , Büchler, M. W. , & Werner, J. (2011). Pancreatic cancer surgery in the new millennium: Better prediction of outcome. Annals of Surgery, 254(2), 311–319. https://10.1097/SLA.0b013e31821fd334 21606835 10.1097/SLA.0b013e31821fd334

[cpz170410-bib-0030] Idorn, M. , & Thor Straten, P. (2017). Exercise and cancer: From ‘healthy’ to ‘therapeutic’? Cancer Immunology, Immunotherapy, 66(5), 667–671. 10.1007/s00262-017-1985-z 28324125 PMC5406418

[cpz170410-bib-0031] Jiang, L. , Ning, D. , & Chen, X. P. (2021). Improvement in distal pancreatectomy for tumors in the body and tail of the pancreas. World Journal of Surgical Oncology, 19(1), 49. 10.1186/s12957-021-02159-9 33588845 PMC7885351

[cpz170410-bib-0032] Kamel, F. H. , Basha, M. A. , Alsharidah, A. S. , & Salama, A. B. (2020). Resistance training impact on mobility, muscle strength and lean mass in pancreatic cancer cachexia: A randomized controlled trial. Clinical Rehabilitation, 34(11), 1391–1399. 10.1177/0269215520941912 32660264

[cpz170410-bib-0033] Karunakaran, M. , & Barreto, S. G. (2021). Surgery for pancreatic cancer: Current controversies and challenges. Future Oncology, 17(36), 5135–5162. 10.2217/fon-2021-0533 34747183

[cpz170410-bib-0034] Kern, K. A. , & Norton, J. A. (1988). Cancer cachexia. Journal of Parenteral and Enteral Nutrition, 12(3), 286–298. 10.1177/0148607188012003286 3292798

[cpz170410-bib-0035] Kim, Y. I. , Song, K. B. , Lee, Y.‐J. , Park, K.‐M. , Hwang, D. W. , Lee, J. H. , Shin, S. H. , Kwon, J. W. , Ro, J.‐S. , & Kim, S. C. (2019). Management of isolated recurrence after surgery for pancreatic adenocarcinoma. British Journal of Surgery, 106(7), 898–909. 10.1002/bjs.11144 31162655

[cpz170410-bib-0036] Kordes, M. , Larsson, L. , Engstrand, L. , & Löhr, J. M. (2021). Pancreatic cancer cachexia: Three dimensions of a complex syndrome. British Journal of Cancer, 124(10), 1623–1636. 10.1038/s41416-021-01301-4 33742145 PMC8110983

[cpz170410-bib-0037] Larson, L. , Degens, H. , & Meishan, L. (2025). Sarcopenia: Aging‐related loss of muscle mass and function. Physiological Reviews, 99(1), 427–511. 10.1152/physrev.00061.2017 PMC6442923 30427277

[cpz170410-bib-0038] Lavalle, S. , Valerio, M. R. , Masiello, E. , Gebbia, V. , & Scandurra, G. (2024). Unveiling the intricate dance: How cancer orchestrates muscle wasting and sarcopenia. In Vivo, 38(4), 1520–1529. 10.21873/invivo.13602 38936901 PMC11215601

[cpz170410-bib-0039] Lee, E. M. , Jiménez‐Fonseca, P. , Galán‐Moral, R. , Coca‐Membribes, S. , Fernández‐Montes, A. , Sorribes, E. , García‐Torralba, E. , Puntí‐Brun, L. , Gil‐Raga, M. , Cano‐Cano, J. , & Calderon, C. (2023). Toxicities and quality of life during cancer treatment in advanced solid tumors. Current Oncology, 30(10), 9205–9216. 10.3390/curroncol30100665 37887565 PMC10605504

[cpz170410-bib-0040] Lee, J. (2022). The effects of resistance training on muscular strength and hypertrophy in elderly cancer patients: A systematic review and meta‐analysis. Journal of Sport and Health Science, 11(2), 194–201. 10.1016/j.jshs.2021.02.002 33592324 PMC9068528

[cpz170410-bib-0041] Lee, J. H. , Gulec, S. A. , Kyshtoobayeva, A. , Sim, M. , & Morton, D. L. (2009). Biological factors, tumor growth kinetics, and survival after metastasectomy for pulmonary melanoma. Annals of Surgical Oncology, 16(10), 2834–2839. 10.1245/s10434-009-0583-5 19603235 PMC2752490

[cpz170410-bib-0042] Liu, J. , Mroczek, M. , Mach, A. , Stępień, M. , Aplas, A. , Pronobis‐Szczylik, B. , Bukowski, S. , Mielczarek, M. , Gajewska, E. , Topolski, P. , Król, Z. J. , Szyda, J. , & Dobosz, P. (2023). Genetics, genomics and emerging molecular therapies of PC. Cancers, 15(3), 779. 10.3390/cancers15030779 36765737 PMC9913594

[cpz170410-bib-0043] Liu, X. , Song, H. , Li, C. , Hu, X. , Li, C. , Lu, G.‐T. , Xiang, H. , & Yu, Q. (2025). Muscle strength and mass as predictors of pancreatic cancer: Insights from the UK biobank. BMC Cancer, 25(1), 1346. 10.1186/s12885-025-14766-w 40836282 PMC12369202

[cpz170410-bib-0044] Liu, Z. , Chen, J. , Ren, Y. , Liu, S. , Ba, Y. , Zuo, A. , Luo, P. , Cheng, Q. , Xu, H. , & Han, X. (2024). Multi‐stage mechanisms of tumor metastasis and therapeutic strategies. Signal Transduction and Targeted Therapy, 9(1), 270. 10.1038/s41392-024-01955-5 39389953 PMC11467208

[cpz170410-bib-0045] Miller, K. D. , Siegel, R. L. , Lin, C. C. , Mariotto, A. B. , Kramer, J. L. , Rowland, J. H. , Stein, K. D. , Alteri, R. , & Jemal, A. (2016). Cancer treatment and survivorship statistics, 2016. Cancer Journal for Clinicians, 66(4), 271–289. 10.3322/caac.21349 27253694

[cpz170410-bib-0046] Owusuaa, C. , Dijkland, S. A. , Nieboer, D. , van der Heide, A. , & van der Rijt, C. C. D. (2022). Predictors of mortality in patients with advanced cancer—a systematic review and meta‐analysis. Cancers, 14(2), 328. 10.3390/cancers14020328 35053493 PMC8774229

[cpz170410-bib-0047] Papadopetraki, A. , Giannopoulos, A. , Maridaki, M. , Zagouri, F. , Droufakou, S. , Koutsilieris, M. , & Philippou, A. (2023). The role of exercise in cancer‐related sarcopenia and sarcopenic obesity. Cancers, 15(24), 5856. 10.3390/cancers15245856 38136400 PMC10741686

[cpz170410-bib-0048] Pawlik, T. M. , Gleisner, A. L. , Cameron, J. L. , Winter, J. M. , Assumpcao, L. , Lillemoe, K. D. , Wolfgang, C. , Hruban, R. H. , Schulick, R. D. , Yeo, C. J. , & Choti, M. A. (2007). Prognostic relevance of lymph node ratio following pancreaticoduodenectomy for PC. Surgery, 141(5), 610–618. 10.1016/j.surg.2006.12.013 17462460

[cpz170410-bib-0049] Qiu, Z. S. , Wang, X. C. , Ma, J. C. , Zhu, C. L. , Hu, Y. L. , & Da, M. X (2025). DEAD/H‐box RNA helicase 10 promotes pancreatic cancer cell proliferation via ribonucleotide reductase M2. World Journal of Gastrointestinal Oncology, 17(9), 108672. https://10.4251/wjgo.v17.i9.108672 40977667 10.4251/wjgo.v17.i9.108672 PMC12444323

[cpz170410-bib-0050] Rawla, P. , Sunkara, T. , & Gaduputi, V. (2019). Epidemiology of pancreatic cancer: Global trends, etiology and risk factors. World Journal of Oncology, 10(1), 10–27. 10.14740/wjon1166 30834048 PMC6396775

[cpz170410-bib-0051] Reed, M. , Patrick, C. , Quevillon, T. , Walde, N. , & Voutsadakis, I. A. (2019). Prediction of hospital admissions and grade 3‐4 toxicities in cancer patients 70 years old and older receiving chemotherapy. European Journal Cancer Care (England), 28(6), e13144. 10.1111/ecc.13144 31429128

[cpz170410-bib-0053] Rosebrock, K. , Sinn, M. , Uzunoglu, F. G. , Bokemeyer, C. , Jensen, W. , & Salchow, J. (2023). Effects of exercise training on patient‐specific outcomes in pancreatic cancer patients: A scoping review. Cancers, 15(24), 5899. 10.3390/cancers15245899 38136443 PMC10741570

[cpz170410-bib-0054] Sarantis, P. , Koustas, E. , Papadimitropoulou, A. , Papavassiliou, A. G. , & Karamouzis, M. V (2020). Pancreatic ductal adenocarcinoma: Treatment hurdles, tumor microenvironment and immunotherapy. World Journal of Gastrointestinal Oncology, 12(2), 173–181. 10.4251/wjgo.v12.i2.173 32104548 PMC7031151

[cpz170410-bib-0055] Schmitz, K. H. , Boutin, R. D. , Yoon, J. P. , Potiaumpai, M. , Lenchik, L. , White, C. M. , & Chang, V. (2023). Exercise intervention during chemotherapy for pancreatic cancer: Changes in body composition and function. JCSM Rapid Communications, 6(1), 33–41. 10.1002/rco2.74

[cpz170410-bib-0056] Schmitz, K. H. , Courneya, K. S. , Matthews, C. , Demark‐Wahnefried, W. , Galvão, D. A. , Pinto, B. M. , Irwin, M. L. , Wolin, K. Y. , Segal, R. J. , Lucia, A. , Schneider, C. M. , von Gruenigen, V. E. , & Schwartz, A. L. (2010). American college of sports medicine roundtable on exercise guidelines for cancer survivors. Medicine & Science in Sports & Exercise, 42(7), 1409–1426. 10.1249/MSS.0b013e3181e0c112 20559064

[cpz170410-bib-0057] Shen, X.‐D. , Wang, X. , Zheng, Z.‐J. , Chen, Y.‐H. , Tan, C.‐L. , Liu, X.‐B. , & Ke, N.‐W. (2023). The differential effects of sarcopenia and cachexia on overall survival for pancreatic ductal adenocarcinoma patients following pancreatectomy: A retrospective study based on a large population. Cancer Medicine, 12(9), 10438–10448. 10.1002/cam4.5779 36938648 PMC10225236

[cpz170410-bib-0059] Spagnolo, C. C. , Giuffrida, G. , Cannavò, S. , Franchina, T. , Silvestris, N. , Ruggeri, R. M. , & Santarpia, M. (2022). Management of endocrine and metabolic toxicities of immune‐checkpoint inhibitors: From clinical studies to a real‐life scenario. Cancers, 15(1), 246. 10.3390/cancers15010246 36612243 PMC9818218

[cpz170410-bib-0061] Sung, H. , Ferlay, J. , Siegel, R. L. , Laversanne, M. , Soerjomataram, I. , Jemal, A. , & Bray, F. (2021). Global cancer statistics 2020: GLOBOCAN estimates of incidence and mortality worldwide for 36 cancers in 185 countries. Cancer Journal for Clinicians, 71(3), 209–249. 10.3322/caac.21660 33538338

[cpz170410-bib-0062] Talbert, E. E. , Cuitiño, M. C. , Ladner, K. J. , Rajasekerea, P. V. , Siebert, M. , Shakya, R. , Leone, G. W. , Ostrowski, M. C. , Paleo, B. , Weisleder, N. , Reiser, P. J. , Webb, A. , Timmers, C. D. , Eiferman, D. S. , Evans, D. C. , Dillhoff, M. E. , Schmidt, C. R. , & Guttridge, D. C. (2019). Modeling human cancer‐induced cachexia. Cell Reports, 28(6), 1612–1622.e4. http://10.1016/j.celrep.2019.07.016 31390573 10.1016/j.celrep.2019.07.016 PMC6733019

[cpz170410-bib-0063] Tan, C. R. , Jamil, L. , Yaffee, P. , Tuli, R. , Nissen, N. , Lo, S. , Nissen, N. , Pandol, S. J. , Tuli, R. , & Hendifar, A. E. (2014). Pancreatic cancer cachexia: A review of mechanisms and therapeutics. Frontiers in Physiology, 5, 88. https://10.3389/fphys.2014.00088 24624094 10.3389/fphys.2014.00088 PMC3939686

[cpz170410-bib-0064] Williams, G. R. , Rier, H. N. , McDonald, A. , & Shachar, S. S. (2019). Sarcopenia & aging in cancer. Journal of Geriatric Oncology, 10(3), 374–377. 10.1016/j.jgo.2018.10.009 30343999 PMC6472991

[cpz170410-bib-0065] Yang, L. , Morielli, A. R. , Heer, E. , Kirkham, A. A. , Cheung, W. Y. , Usmani, N. , Friedenreich, C. M. , & Courneya, K. S. (2021). Effects of exercise on cancer treatment efficacy: A systematic review of preclinical and clinical studies. Cancer Research, 81(19), 4889–4895. 10.1158/0008-5472.CAN-21-1258 34215623 PMC9397632

[cpz170410-bib-0066] Yoh, K. , Nishikawa, H. , Enomoto, H. , Ishii, N. , Iwata, Y. , Ishii, A. , Yuri, Y. , Miyamoto, Y. , Hasegawa, K. , Nakano, C. , Takata, R. , Nishimura, T. , Aizawa, N. , Sakai, Y. , Ikeda, N. , Takashima, T. , Iijima, H. , & Nishiguchi, S. (2018). Effect of exercise therapy on sarcopenia in pancreatic cancer: A study protocol for a randomised controlled trial. BMJ Open Gastroenterology, 5(1), e000194. 10.1136/bmjgast-2017-000194 PMC5841516 29527315

